# Reduced alcohol consumption during the COVID-19 pandemic: Analyses of 17 000 patients seeking primary health care in Colombia and Mexico

**DOI:** 10.7189/jogh.12.05002

**Published:** 2022-03-19

**Authors:** Jakob Manthey, Sinclair Carr, Peter Anderson, Natalia Bautista, Fleur Braddick, Amy O’Donnell, Eva Jané-Llopis, Hugo López-Pelayo, Perla Medina, Juliana Mejía-Trujillo, Augusto Pérez-Gómez, Marina Piazza, Jürgen Rehm, Adriana Solovei, Guillermina Natera Rey, Hein de Vries, Bernd Schulte

**Affiliations:** 1 Institute of Clinical Psychology and Psychotherapy, Technische Universität Dresden, Dresden, Germany; 2 Center for Interdisciplinary Addiction Research (ZIS), Department of Psychiatry and Psychotherapy, University Medical Center Hamburg, Hamburg, Germany; 3 Department of Psychiatry, Medical Faculty, University of Leipzig, Leipzig, Germany; 4 Heidelberg Institute of Global Health, Faculty of Medicine and University Hospital, Heidelberg University, Heidelberg, Germany; 5 Department of Health Promotion, CAPHRI Care and Public Health Research Institute, Maastricht University, Maastricht, the Netherlands; 6 Population Health Sciences Institute, Newcastle University, Newcastle upon Tyne, UK; 7 Instituto Nacional de Psiquiatría Ramón de la Fuente Muñiz, Huipulco, Ciudad de México, CDMX, Mexico; 8 Addictions Unit, Psychiatry Dept, Hospital Clínic, Barcelona, Spain; 9 Univ. Ramon Llull, ESADE, Barcelona, Spain; 10 Institute for Mental Health Policy Research, CAMH, Toronto, Canada; 11 Red de Trastornos Adictivos, Instituto Carlos III, Madrid, Spain; 12 Institut d’Investigacions Biomèdiques August Pi Sunyer (IDIBAPS), Barcelona, Spain; 13 Corporación Nuevos Rumbos, Bogotá, Colombia; 14 School of Public Health and Administration, Universidad Peruana Cayetano Heredia, Lima, Peru; 15 Dalla Lana School of Public Health, University of Toronto, Toronto, Ontario, Canada; 16 Campbell Family Mental Health Research Institute, Centre for Addiction and Mental Health, Toronto, Ontario, Canada; 17 Department of Psychiatry, University of Toronto, Toronto, Ontario, Canada; 18 Department of International Health Projects, Institute for Leadership and Health Management, I.M. Sechenov First Moscow State Medical University, Moscow, Russian Federation; 19 Agència de Salut Pública de Catalunya, Barcelona, Spain

## Abstract

**Background:**

During the COVID-19 pandemic, an increase of heavy alcohol use has been reported in several high-income countries. We examined changes in alcohol use during the pandemic among primary health care (PHC) patients in two middle income countries, Colombia and Mexico.

**Methods:**

Data were collected during routine consultations in 34 PHC centres as part of a large-scale implementation study. Providers measured patients’ alcohol consumption with the three item ‘Alcohol Use Disorders Identification Test’ (AUDIT-C). Generalized linear mixed models were performed to examine changes in two dependent variables over time (pre-pandemic and during pandemic): 1) the AUDIT-C score and 2) the proportion of heavy drinking patients (8+ on AUDIT-C).

**Results:**

Over a period of more than 600 days, data from N = 17 273 patients were collected. During the pandemic, the number of patients with their alcohol consumption measured decreased in Colombia and Mexico. Each month into the pandemic was associated with a 1.5% and 1.9% reduction in the mean AUDIT-C score in Colombia and Mexico, respectively. The proportion of heavy drinking patients declined during the pandemic in Colombia (pre-pandemic: 5.4%, 95% confidence interval (CI) = 4.8% to 6.0%; during the pandemic: 0.8%, 95% CI = 0.6% to 1.1%) but did not change in Mexico.

**Conclusions:**

Average consumption levels declined and the prevalence of heavy drinking patterns did not increase. In addition to reduced opportunities for social drinking during the pandemic, changes in the population seeking PHC and restrictions in alcohol availability and affordability are likely drivers for lower levels of alcohol use by patients in this study.

Alcohol consumption causes considerable disease burden as well as economic and social costs [1,2]. In low- and middle-income countries, the burden from alcohol consumption is disproportionately higher [3] and alcohol consumption levels have increased rapidly over the recent decades as compared to high-income countries. In Latin America, alcohol is the 4th largest risk factor for morbidity, compared to being the 9th globally [4].

Since March 2020, the Severe Acute Respiratory Syndrome Coronavirus 2 spread rapidly throughout global populations reaching pandemic status, with unprecedented and profound impacts on all aspects of life. Existing literature points to two important developments relevant to alcohol consumption and attributable harm.

First, survey data indicate that whilst there have been moderate changes in overall alcohol consumption during the global Coronavirus Disease 2019 (COVID-19) pandemic (see eg, [5,6]), a polarisation of drinking practices seems to have taken place. This polarisation is reflected in increased consumption amongst existing high-risk alcohol users (ie, those already drinking at excessive levels prior to COVID-19) and decreased consumption among low-risk alcohol users (see eg, [7,8]). Furthermore, an increase in adverse outcomes linked to very heavy drinking has been identified, including alcohol withdrawal treatment and liver disease presentations. While the bulk of evidence was collected in high-income countries, a large-scale survey covering 33 Latin American and Caribbean countries also suggests an overall decrease of heavy episodic drinking during this period [9].

Second, a priority shift in delivering health care services due to the global COVID-19 pandemic has been observed. The rapid spread of COVID-19 has overwhelmed health care systems worldwide as they struggled to manage health resources (ie, staff, hospital beds, etc.) in order to prevent overload and saturation [10]. The pandemic impact has not been restricted to secondary care, however, as reflected in considerable reductions in the number of primary health care (PHC) contacts documented in several high-income and middle-income countries.

The observed polarisation of alcohol use, in particular the increased heavy alcohol use in existing heavy drinkers, coupled with a reduced health care access during the pandemic are cause for concern. To improve our understanding of changes in alcohol consumption during the pandemic, especially in middle-income countries, we analysed data from a large-scale quasi-experimental study conducted in PHC settings in Mexico and Colombia. Real-world data from 34 primary health care centres (PHCCs) in Colombia and Mexico were used to assess changes in alcohol use patterns during the COVID-19 pandemic among PHC patients who have their alcohol consumption measured. Specifically, we posed the following two hypotheses, which are based on the observed polarisation of alcohol use during the pandemic:

1. On average, alcohol consumption has decreased with the onset of the COVID-19 pandemic.
2. The proportion of patients reporting heavy drinking has not decreased with the onset of the COVID-19 pandemic.

## METHODS

This study follows the ‘Strengthening the Reporting of Observational studies in Epidemiology’ (STROBE) guidelines [11]. The completed STROBE checklist is included in Table S1 in the **Online Supplementary Document**.

### Study design and sample

The international SCALA project (‘Scale-up of Prevention and Management of Alcohol Use Disorders and Comorbid Depression in Latin America’, www.scalaproject.eu) is a quasi-experimental study evaluating the impact of training and community support on rates of alcohol measurement and brief advice for heavy drinking patients among PHC providers in Colombia, Mexico, and Peru, as described in the protocol and primary outcome paper [12]. Analyses for this paper were restricted to data collected in Colombia (Soacha, Funza and Madrid municipalities in Cundinamarca region) and Mexico (Tllapan, Benito Juarez, Álvaro Obregón, Miguel Hidalgo and Xochimilco municipalities in Mexico City), as data collection in Peru came to a complete halt due to COVID disruption of PHC visits in Peru, and only resumed during May 2021.

### Measures

For the current analyses, we used tally sheet data completed by N = 220 PHC providers during the implementation phase of the project (20 August 2019 to 30 April 2021). According to the study protocol, all consulting adults visiting one of the PHCCs should be offered an alcohol measurement during their consultation, which was to be documented using the tally sheet. Previous analyses on the same data, however, suggested that measurement rates differed between PHCCs [12].

Data obtained from tally sheets included responses to the short form of the ‘Alcohol Use Disorder Identification Test’ (AUDIT-C), in addition to information on gender (male, female, other), age (grouped in 18-29, 30-39, 40-49, 50-59, 60-69, 70+ years), and education level (any education higher than high school, high school, lower education than high school).

Lastly, we obtained information on gender, age, and profession (doctor, nurse, technician nurse, midwife, social worker, psychologist, or other) of all health care providers from a questionnaire completed prior to study participation.

### Data definitions

To test the two hypotheses, we analysed two dependent variables: 1) the AUDIT-C score (sum of the three items, range: 0-12) and 2) the proportion of heavy drinking patients, defined as an AUDIT-C score 8+. Changes in the two dependent variables over time were analysed using both a continuous and a dummy indicator.

For the first indicator, we used a continuous time variable that was centered at the start of the pandemic (for the definition, see next paragraph) and scaled in months so that negative values indicated the number of months before the pandemic and positive values indicated the number of months into the pandemic. In Colombia, data collection paused shortly after the begin of the pandemic and lasted for a period of 152 days. To avoid making assumptions on trends for a period in which no data was available in Colombia, we removed this period from the data. Thus, in Colombia, the data collection period *during* the pandemic was shifted to start five rather than 152 days after the last AUDIT-C sheet had been collected *prior* to the start of the pandemic.

For the second indicator, the study period was split into a pre-pandemic and a during pandemic period. The date of onset of the COVID-19 pandemic was defined when measures to contain the spread of the virus were implemented in each country. In Colombia, the so-called state of economic, social, and ecological emergency was declared by the president on March 17, 2020 along with the first obligatory measures [13,14]. In Mexico, the so called ‘National Day of Healthy Distance’ was implemented by the Mexican Government on March 23, 2020, focusing on recommending social distancing measures including to abstain from alcohol use [15,16]. In both countries, these dates coincided with very low but increasing numbers of COVID-19 infections.

### Statistical analyses

The analytical sample consisted of all tally sheets containing at least one response on the AUDIT-C, including both abstainers and drinkers. χ2 tests were performed for comparison of all patient characteristics’ variables before and during the pandemic, with additional *t* test for age as a continuous variable. Country-specific models were conducted because the data collection periods were not comparable in the two countries and the COVID-19 pandemic was not assumed to have impacted the two countries in the same way.

To assess changes in the alcohol profile of documented patients, generalized linear mixed models were conducted. Accounting for the distribution of the dependent variable, negative binomial models were performed for the AUDIT-C score and logistic models were performed for the proportion of patients scoring 8+ on the AUDIT-C. Each model was stratified by country and adjusted for gender, age group and profession of the PHC provider, in addition to the patient gender, age group and education level.

Further, the clustered structure of the data was considered in the models by including random effects for each provider, which was nested within each PHCC. The random intercepts also allowed to control for variations in alcohol measurement behavior across providers and PHCCs. For the second dependent variable (% of patients scoring 8+ on the AUDIT-C), the regression models showed indicators of singularity for both countries. To avoid overfitting the model using Colombian data, we removed the nested structure of the random effects, ie, the assignment of providers to PHCCs. To avoid overfitting the model using Mexican data, we removed the nested random effects structure in addition to excluding n = 44 observations without documented patient gender as none of them scored 8+ on the AUDIT-C. Post-hoc analyses of changes in alcohol consumption related to the pandemic were performed by age group and education. Rather than performing additional regression models stratified by age and education, we compared average AUDIT-C responses before and during the pandemic for select age and educational groups and meaningful changes were identified by non-overlapping 95% confidence intervals. This approach was prefered because of low sample sizes in stratified groups and because this is a simple and conservative approach which also avoids to cumulate alpha errors due to multiple testing.

All statistical analyses were performed using R Version 4.0.5 [17]. The R code and all data analysed for this paper can be found elsewhere (https://figshare.com/s/2a5a858902db29b59001).

Ethics approval: The study was approved by the Ethics Committee of the Technical University of Dresden (EK90032018) and all participating PHC providers have signed an informed consent form prior to study participation.

## RESULTS

### Sample characteristics

Over a period of 619 and 602 days, 10 659 and 6614 alcohol measurements were performed in Colombia and Mexico respectively (see **Table 1**).

**Table 1:** Sample description*

Country	Colombia		Mexico	
**Period**	**Before**	**During**	**Before**	**During**
Days of data collection	148	163	163	285
**Providers:**				
N	62	8	116	34
Gender:				
Women	74.2% (61.6-83.8)	87.5% (31.9-99.1)	71.6% (62.5-79.1)	61.8% (43.8-77.0)
Men	25.8% (16.2-38.4)	12.5% (0.9-68.1)	28.4% (20.9-37.5)	38.2% (23.0-56.2)
Age:†				
17-29	51.6% (39.0-64.0)	62.5% (20.8-91.3)	31.9% (24.0-41.0)	14.7% (6.0-31.9)
30-39	19.4% (11.2-31.5)	12.5% (0.9-68.1)	35.3% (27.1-44.6)	41.2% (25.4-59.0)
40-49	16.1% (8.7-27.8)	25.0% (4.1-72.4)	16.4% (10.6-24.4)	14.7% (6.0-31.9)
50-59	6.5% (2.4-16.4)	0.0% (0.0-0.0)	5.2% (2.3-11.2)	11.8% (4.3-28.6)
60-69	3.2% (0.8-12.4)	0.0% (0.0-0.0)	7.8% (4.0-14.4)	2.9% (0.4-19.8)
70-99	0.0% (0.0-0.0)	0.0% (0.0-0.0)	0.9% (0.1-6.0)	2.9% (0.4-19.8)
Profession:				
Doctor	51.6% (39.0-64.0)	75.0% (27.6-95.9)	65.5% (56.3-73.7)	50.0% (33.0-67.0)
Nurse (technician)	38.7% (27.2-51.7)	25.0% (4.1-72.4)	7.8% (4.0-14.4)	2.9% (0.4-19.8)
Midwife/social worker	1.6% (0.2-11.1)	0.0% (0.0-0.0)	0.9% (0.1-6.0)	5.9% (1.4-22.0)
Psychologist	1.6% (0.2-11.1)	0.0% (0.0-0.0)	7.8% (4.0-14.4)	32.4% (18.3-50.5)
Other/not reported	6.5% (2.4-16.4)	0.0% (0.0-0.0)	18.1% (12.0-26.3)	8.8% (2.7-25.2)
**Patients:**				
N	5787	4872	3569	3045
Gender:‡				
Female	74.0% (72.8-75.1)	78.6% (77.4-79.7)	62.0% (60.4-63.6)	57.4% (55.7-59.2)
Male	24.6% (23.5-25.8)	19.7% (18.6-20.8)	37.6% (36.1-39.2)	41.5% (39.8-43.3)
Other/not reported	1.4% (1.1-1.7)	1.7% (1.4-2.2)	0.4% (0.2-0.6)	1.0% (0.7-1.4)
Age:‡,§				
18-29	29.3% (28.2-30.5)	29.0% (27.7-30.3)	25.7% (24.3-27.2)	26.7% (25.2-28.3)
30-39	16.3% (15.4-17.3)	14.1% (13.1-15.1)	19.3% (18.0-20.6)	22.5% (21.1-24.0)
40-49	14.9% (14.0-15.8)	12.2% (11.3-13.1)	17.9% (16.7-19.2)	19.1% (17.8-20.6)
50-59	15.6% (14.6-16.5)	17.1% (16.0-18.2)	17.5% (16.3-18.8)	16.5% (15.2-17.8)
60-69	12.3% (11.4-13.1)	18.2% (17.1-19.3)	12.2% (11.2-13.3)	9.5% (8.5-10.6)
70-99	9.7% (9.0-10.5)	8.8% (8.0-9.6)	6.7% (5.9-7.5)	4.9% (4.2-5.8)
Education:‡				
Any education beyond high school	6.5% (5.9-7.2)	5.8% (5.2-6.5)	17.3% (16.1-18.5)	27.6% (26.0-29.2)
High school	42.5% (41.2-43.8)	51.7% (50.3-53.1)	30.0% (28.5-31.5)	36.4% (34.7-38.1)
Lower education than high school	48.5% (47.2-49.8)	41.1% (39.7-42.5)	52.0% (50.4-53.6)	34.9% (33.2-36.6)
Not reported	2.5% (2.2-3.0)	1.4% (1.1-1.8)	0.8% (0.5-1.1)	1.2% (0.9-1.7)

*Note. For patient variables, group (mean) comparisons before and during the pandemic were conducted. Numbers in brackets represent 95% confidence intervals.

†n = 9 missing values.

‡Groups differed (*P* < 0.001) before and during the pandemic in χ^2^-tests in Colombia and in Mexico.

§n = 193 missing values.

With the onset of the COVID-19 pandemic, the number of PHC workers measuring the alcohol consumption of their patients decreased in Colombia and Mexico. In both countries, most providers in this sample were female and under 40 years old. Although there were almost no psychologists in Colombia and very few in Mexico before the COVID-19 pandemic, they accounted for about one-third of all providers during the pandemic in Mexico.

Comparing patient characteristics before and during the pandemic revealed contrasting patterns in Colombia and Mexico. Whilst the share of women increased in Colombia, it declined in Mexico (results from χ^2^ tests, *P* < 0.001). *t* tests further suggest that during the pandemic, patients with their alcohol consumption measured were on average older in Colombia (44.8 vs 43.6 years, *P* = 0.001) and younger in Mexico (43.2 vs 41.8 years, *P* < 0.001). Lastly, the proportion of patients with high educational attainment decreased in Colombia but increased in Mexico (results from χ^2^ tests, *P* < 0.001).

In both countries, the number of PHC patients whose alcohol consumption was measured dropped considerably at around the dates defined as the start of the COVID-19 pandemic (see Figure S1 in the **Online Supplementary Document**). In Colombia, no tally sheets were completed between 19 March and 20 August 2020, as mentioned above. In Mexico, a considerably lower number of patients were documented between March and August 2020 as compared to the pre-pandemic study period. In both countries, data collection resumed to roughly previous levels in September 2020.

### Hypothesis 1: Changes in mean alcohol consumption

**Figure 1:**
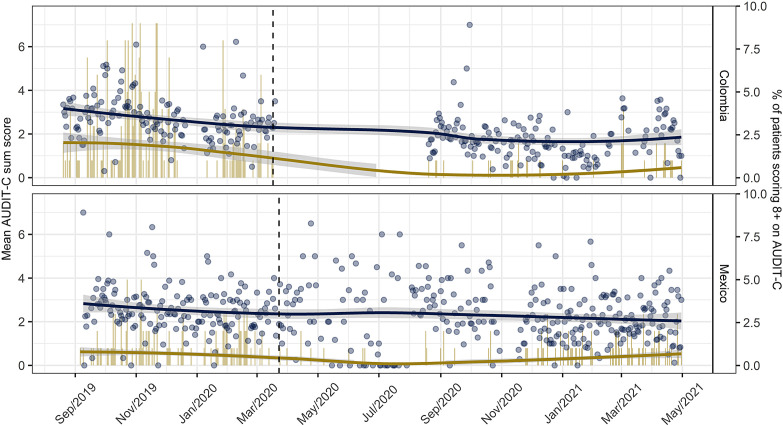
Daily data on the alcohol profile of documented primary health care patients in Colombia and Mexico. Blue circles and smoothed lines indicate mean AUDIT-C score (left y-axis). Yellow bars and smoothed lines indicate the proportion of measured patients scoring 8+ on the AUDIT-C (right y-axis). Smoothed lines and shades (confidence interval) was obtained from local smoothing function (LOESS). The dashed vertical lines indicate the onset of the COVID-19 pandemic in Colombia (17 March, 2020) and Mexico (March 23, 2020).

As illustrated in **Figure 1**, the mean AUDIT-C score of documented patients decreased in both countries over the study period. In Colombia, the mean AUDIT-C score decreased from 2.7 (95% confidence interval (CI) = 2.6 to 2.7) to 1.9 (95% CI = 1.9 to 2.0) when comparing periods before and during the pandemic. In Mexico, the mean AUDIT-C score of documented patients decreased from 2.5 (95% CI = 2.4 to 2.5) to 2.1 (95% CI = 2.0 to 2.2). Accounting for changes in provider and patients’ characteristics, results from mixed-effects negative binomial regression analyses confirmed significant decreases of the AUDIT-C score to a similar extent in both countries (see **Table 2** and Table S2 in the **Online Supplementary Document**). In Colombia and Mexico, each successive month into the pandemic was associated with a 1.5% and 1.9% reduction in the mean AUDIT-C score, respectively. Correspondingly, AUDIT-C scores of documented patients were 21% lower after 17 March 2020 in Colombia and 14% lower after 23 March in Mexico.

**Table 2:** Selected results from mixed-effects regression analyses

Dependent variable	AUDIT-C score†		% of patients scoring 8+ on AUDIT-C‡	
**Country**	**Colombia**	**Mexico**	**Colombia**	**Mexico**
N	10 658	6613	10 444	6569
**Monthly trend** (Changes associated with each additional month into the pandemic§)	0.985 (0.979-0.992)*	0.981 (0.975-0.986)*	0.89 (0.85-0.94)*	0.98 (0.96-1.01)
**Period effect** (Reference period: data collected prior the pandemic onset)	0.79 (0.76-0.84)*	0.86 (0.80-0.92)*	0.41 (0.26-0.64)*	0.81 (0.55-1.20)

AUDIT-C – Alcohol Use Disorder Identification Test – Consumption

*P ≤ 0.001

†Negative binomial mixed-effects regression analyses, with random intercepts for providers clustered within primary health care practices. Presented are Incidence Rate Ratios, ie, exponentiated coefficients. Numbers in brackets indicate Wald-based confidence intervals.

‡Logistic mixed-effects regression analyses, with random intercepts for providers clustered within primary health care practices. Presented are Odds Ratios, ie, exponentiated coefficients. Numbers in brackets indicate Wald-based confidence intervals.

§Centered at beginning of COVID-19 pandemic.

**Figure 2:**
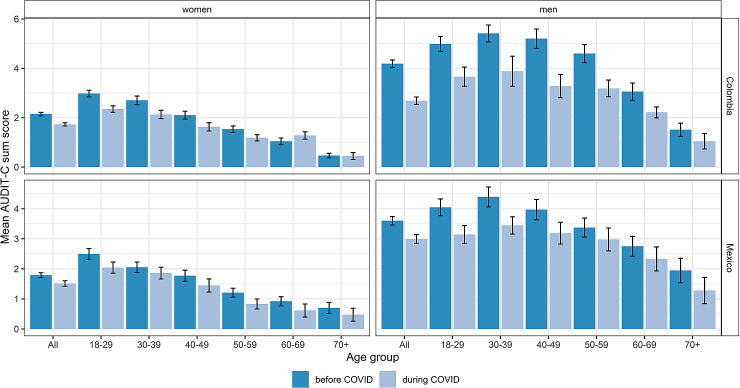
Average alcohol use before and during the pandemic among documented primary health care patients in Colombia and Mexico by sex and age group. Legend: Bars indicate mean AUDIT-C score separately for the period before and during the COVID-19 pandemic (onset in Colombia: 17 March, 2020; in Mexico: March 23, 2020).

As illustrated in **Figure 2**, the decreases in mean AUDIT-C score were seen across all age groups but more pronounced in younger patients, as indicated by non-overlapping confidence intervals of mean scores obtained from patients in the same age group pre- and during the pandemic. In Colombia, meaningful reductions in drinking levels were identified in men aged18 to 69 and women aged 18 to 59 years. In Mexico, meaningful reductions were observed in men aged18 to 49 years, as well as in women in 18-29 and 50-59 age groups.

**Figure 3:**
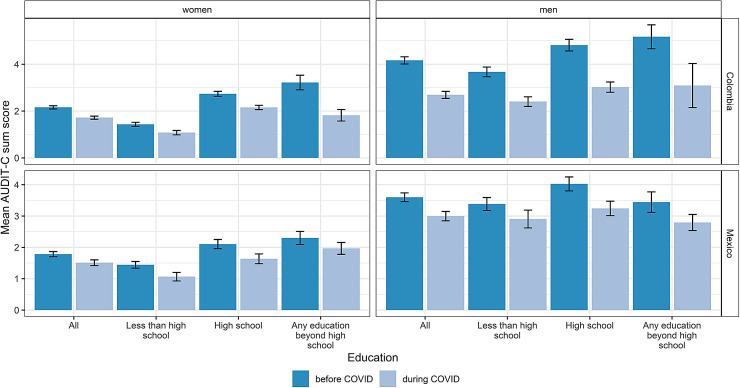
Average alcohol use before and during the pandemic among documented primary health care patients in Colombia and Mexico by sex and educational achievement. Bars indicate mean AUDIT-C score separately for the period before and during the COVID-19 pandemic (onset in Colombia: 17 March, 2020; in Mexico (March 23, 2020)).

As illustrated in **Figure 3**, the decreases in mean AUDIT-C score were quite evenly distributed across educational achievement, as indicated by non-overlapping confidence intervals of mean scores obtained from patients in the same educational group pre- and during the pandemic. In Colombia, meaningful reductions in drinking levels were identified across all educational groups. In Mexico, higher and middle educated men, as well as lower and middle educated women were observed to show meaningful reductions in drinking levels.

### Hypothesis 2: Changes in the proportion of patients reporting heavy drinking

The percentage of documented patients scoring 8+ on the AUDIT-C declined in Colombia (pre-pandemic: 5.4%, 95% CI = 4.8% to 6.0%; during the pandemic: 0.8%, 95% CI = 0.6% to 1.1%), while no changes were registered in Mexico (pre-pandemic: 3.3%, 95% CI = 2.7% to 3.9%; during the pandemic: 3.2%, 95% CI = 2.6% to 3.9%). The regression analyses corroborated this pattern, suggesting that with every additional month into the pandemic, the odds for a documented patient in Colombia to score 8+ on the AUDIT-C decreased by about 11%. Correspondingly, the odds for a documented patient to score 8+ on the AUDIT-C was on average 61% lower after 17 March 2020. In Mexico, the odds of patients scoring 8+ on the AUDIT-C did not significantly vary over time (see **Table 2** and Table S3 in the **Online Supplementary Document**). A more detailed illustration of changes in alcohol consumption is presented and described in Figure S2 in the **Online Supplementary Document**.

**Figure 4:**
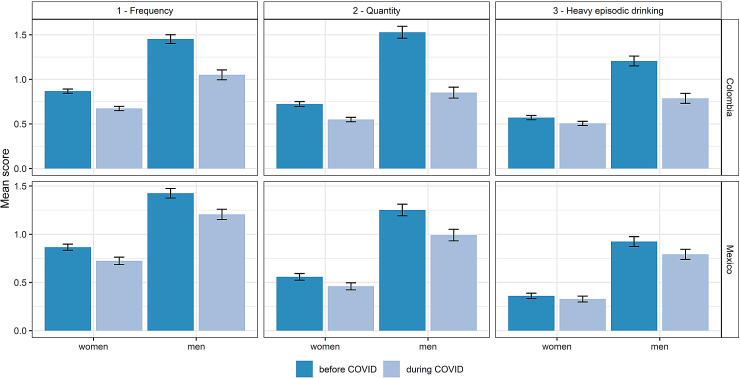
Average alcohol use before and during the pandemic among documented primary health care patients in Colombia and Mexico by sex and AUDIT-C item. Bars indicate mean AUDIT-C score on each item (1: Frequency of alcohol use; 2: Quantity of alcohol on use days; 3: Frequency of heavy episodic drinking) separately for the period before and during the COVID-19 pandemic (onset in Colombia: 17 March, 2020; in Mexico (March 23, 2020)).

To corroborate changes in heavy alcohol use, we examined changes in drinking patterns, ie, in frequency and quantity of alcohol use, as well as heavy episodic drinking. As illustrated in **Figure 4**, meaningful reduction, as indicating by non-overlapping confidence intervals, were observed for all three AUDIT-C items in Colombian men and women. A similar pattern was observed for Mexican men, however, meaningful reductions in the frequency of heavy episodic drinking (AUDIT item 3) could not be identified for Mexican women.

## DISCUSSION

### Main findings

In this study, we analysed AUDIT-C test scores from approximately 17 000 PHC patients collected over a period of nearly two years in Colombia and Mexico, including during the first year of the global COVID-19 pandemic. To our knowledge, this is the first analysis of routine alcohol management data collected in PHC settings during the COVID-19 pandemic.

Based on our analyses, we found support for our first hypotheses, stating a reduction in mean drinking levels among consulting primary health care patients. For the second hypothesis, however, we found conflicting patterns: while the share of heavy drinking patients largely declined in Colombia, no such changes were apparent in Mexico. While reductions in alcohol use appeared to be slightly more pronounced in Colombia (-21%) as compared to Mexico (-14%), we observed meaningful declining drinking levels mostly independent of patient age group or educational achievement.

### Explanations for reduced alcohol use

Our findings largely parallel results from existing online surveys of alcohol consumption during the COVID-19 pandemic, suggesting stronger declines in drinking in Colombia [18] than in Mexico [19] (for an international review of studies, see eg, [20]). As a key driver for reduced alcohol consumption during the pandemic, restrictions to social gatherings have been suggested, both in terms of closed bars, venues and events and acceptable social contact with colleagues, friends and family [21]. For the majority of people who drink, social gatherings are key to engage in drinking and a lack of such occasions may result in lower mean drinking levels as demonstrated in this study. While previous studies showed that average declines in drinking levels may mask increases among heavy drinking subpopulations (see eg, [22]), we did not find similar patterns. There are several potential explanations for a non-increasing prevalence of heavy drinking patterns found in this study.

First, the data analysed here refer to patients seeking PHC services before and during the pandemic and to some degree, the observed changes in drinking patterns are likely to reflect changes in the type of patients attending PHCCs. Whilst we accounted for changes in the sociodemographic composition of both providers and patients in our analyses, we could not account for unobserved changes in the characteristics of the population seeking PHC. For example, health care provision in general may have been restricted to patients with severe health complications during this period than prior to the pandemic. As these patients are usually advised to abstain from drinking alcohol, this could explain lower drinking levels observed in this study. Moreover, people with alcohol use disorders are at increased risk for COVID-19 transmission, hospitalisation, and mortality [23,24]. Considering that this group of drinkers are more often and more severely affected by COVID-19, they would require more intensive care and would thus be less likely to seek PHC services.

In addition to changes in the composition of patients seeking PHC, the prevalence of heavy drinking may also be impacted by restrictive alcohol policy measures enacted during the pandemic. In Colombia, limits for purchasing and consumption of alcoholic beverages were in place between March and September 2020 and the consumption of alcohol continued to be forbidden in public places, bars and restaurants until November [14]. Further measures to restrict on-site consumption and purchases, depending on the local health burden from COVID-19 followed. In Bogotá, for example, alcohol purchases were mostly limited to online or phone orders in April 2021 [25]. In Mexico, several alcohol control measures have been implemented throughout the year 2020, resulting in temporary suspension of beer production and restriction of alcohol sales [26].

To date, the effects of these restrictive set of alcohol control policies have not been evaluated. However, it is reasonable to assume that reduced availability of alcoholic beverages has contributed to a decline in drinking levels, albeit more so amongst low-risk than high-risk drinkers. Lastly, economic losses may have decreased affordability of alcoholic beverages and further driven declines in consumption.

An important aspect that we were unable to examine in this study is related to possible changes in the acquisition of alcohol. In Colombia and Mexico, the consumption of alcohol from unregistered sources is prevalent and was estimated to constitute about 20% of all alcohol consumed [27]. Possibly, the closure of licensed retail outlets during the COVID-19 pandemic may drive heavy and dependent drinkers to resort to illicit sources or surrogate alcohol.

### Impact of COVID on alcohol management in primary health care settings

In addition to changes in alcohol consumption, we found a drop in the documentation of alcohol consumption among consulting PHC patients after the onset of the pandemic. According to our data, no patient had their alcohol consumption measured for almost 22 weeks in the Colombian PHCCs participating in this study, and the number of professionals measuring alcohol use throughout the pandemic period decreased substantially. The shift of priorities towards tackling the COVID-19 pandemic, as also observed in our study, has disrupted health care systems and in particular PHC services [28]. In Colombia, PHCCs were almost entirely suspended for several months and providers were asked to manage COVID-19 cases regardless of their specialisation [29,30]. In contrast, PHC facilities remained open in Mexico and they played a key role to mitigate the pandemic [31], however, the number of consulting patients also declined here.

In order to understand why some providers continued to prioritise alcohol management in their everyday work during the COVID-19 pandemic, additional in-depth analyses are required and planned [32]. Identifying drivers and facilitators of continued alcohol measurement in times of strained resources or public health crises may be particularly valuable to inform future efforts to scale-up alcohol management activities in PHC settings, particularly in low and middle-income countries.

### Limitations

There are some limitations, which should be considered when interpreting the results. First, we acknowledge that the sampled patients are not representative of the adult population in the two countries. Utilization of health care services depends strongly on gender, age and socioeconomic status [33] and this bias may be even more pronounced during the COVID-19 pandemic. However, the average AUDIT-C scores observed in this study are what one would expect based on drinking levels in Colombia and Mexico. For comparison, mean AUDIT-C estimates ranged between 2.6 and 3.2 in a sample of German primary health care patients (unpublished data from our group). The on average lower AUDIT-C values in Colombia and Mexico may reflect that per capita alcohol consumption is higher in Germany as compared to Colombia and Mexico. Moreover, the distribution of AUDIT-C scores as illustrated in Figure S2 in the **Online Supplementary Document** follows the classic pattern of alcohol consumption: most people drink lower amounts (eg, AUDIT-C scores 1 to 7) and few people drink larger amounts (eg, AUDIT-C scores 8 to 12).

Second, we could not assess whether alcohol measurement was biased towards certain populations and could not quantify how the population seeking PHC services has changed during the pandemic. While we have attempted to control for possibly confounding variables, including provider and patient characteristics, we cannot rule out that the observed changes in alcohol consumption are related to unobserved changes in the composition of the patient population rather than to actual changes in alcohol consumption. Furthermore, we cannot rule out that the COVID pandemic has changed how patients remember and/or report their alcohol consumption. Lastly, we analysed data from urban centres of the two countries. A generalization of these findings to the entire country may be limited by possible urban-rural differences in the sociodemographic composition of the population, which is a known driver for variation in alcohol use and attributable harm [34].

## CONCLUSIONS

With the onset of the COVID-19 pandemic, the number of alcohol measurements performed in PHC settings in Colombia and Mexico declined due to decreased patient attendance. Amongst patients who had their alcohol consumption measured, average consumption levels declined and the prevalence of heavy drinking patterns did not increase, in contrast to findings of polarised drinking patterns in other parts of the world. In addition to reduced opportunities for social drinking during the pandemic, changes in the population seeking PHC services and changes in alcohol availability and affordability may account for lower alcohol use levels among patients in this study.

## Additional material


Online Supplementary Document
