# Supplementary material for: Reduced alcohol consumption during the COVID-19 pandemic: Analyses of 17 000 patients seeking primary health care in Colombia and Mexico
Source: J Glob Health. 2022 Mar 19;12:05002. doi: 10.7189/jogh.12.05002 (PMC8932607; doi:10.7189/jogh.12.05002)
Supplement: Online Supplementary Document [file jogh-12-05002-s001.pdf]

Online Supplementary Document

Title: Reduced alcohol consumption during the COVID-19 pandemic: Analyses of 17,000 patients seeking primary health care in Colombia and Mexico

Authors:

Jakob Manthey, Sinclair Carr, Peter Anderson, Natalia Bautista, Fleur Braddick, Amy O'Donnell, Eva Jané-Llopis, Hugo López-Pelayo, Perla Medina, Juliana Mejía-Trujillo, Augusto Pérez-Gómez, Marina Piazza, Jürgen Rehm, Adriana Solovei, Guillermina Natera Rey, Hein de Vries, Bernd Schulte

Table S1. 'Strengthening the Reporting of Observational studies in Epidemiology' (STROBE) Checklist

	Item No	Recommendation	Page No
Title and abstract	1	(*a*) Indicate the study's design with a commonly used term in the title or the abstract	1
		(*b*) Provide in the abstract an informative and balanced summary of what was done and what was found	1
**Introduction**			
Background/rationale	2	Explain the scientific background and rationale for the investigation being reported	2-3
Objectives	3	State specific objectives, including any prespecified hypotheses	3
**Methods**			
Study design	4	Present key elements of study design early in the paper	4
Setting	5	Describe the setting, locations, and relevant dates, including periods of recruitment, exposure, follow-up, and data collection	4
Participants	6	(*a*) Give the eligibility criteria, and the sources and methods of selection of participants	4
Variables	7	Clearly define all outcomes, exposures, predictors, potential confounders, and effect modifiers. Give diagnostic criteria, if applicable	4-5
Data sources/ measurement	8*	For each variable of interest, give sources of data and details of methods of assessment (measurement). Describe comparability of assessment methods if there is more than one group	4-5
Bias	9	Describe any efforts to address potential sources of bias	-
Study size	10	Explain how the study size was arrived at	4
Quantitative variables	11	Explain how quantitative variables were handled in the analyses. If applicable, describe which groupings were chosen and why	4-5

Statistical methods	12	(*a*) Describe all statistical methods, including those used to control for confounding	6
		(*b*) Describe any methods used to examine subgroups and interactions	-
		(*c*) Explain how missing data were addressed	6
		(*d*) If applicable, describe analytical methods taking account of sampling strategy	-
		(*e*) Describe any sensitivity analyses	-
**Results**			
Participants	13*	(a) Report numbers of individuals at each stage of study—eg numbers potentially eligible, examined for eligibility, confirmed eligible, included in the study, completing follow-up, and analysed	7
		(b) Give reasons for non-participation at each stage	-
		(c) Consider use of a flow diagram	-
Descriptive data	14*	(a) Give characteristics of study participants (eg demographic, clinical, social) and information on exposures and potential confounders	7
		(b) Indicate number of participants with missing data for each variable of interest	Tab. 1
Outcome data	15*	Report numbers of outcome events or summary measures	8
Main results	16	(*a*) Give unadjusted estimates and, if applicable, confounder-adjusted estimates and their precision (eg, 95% confidence interval). Make clear which confounders were adjusted for and why they were included	8
		(*b*) Report category boundaries when continuous variables were categorized	5
		(*c*) If relevant, consider translating estimates of relative risk into absolute risk for a meaningful time period	-

Other analyses	17	Report other analyses done—eg analyses of subgroups and interactions, and sensitivity analyses	-
**Discussion**			
Key results	18	Summarise key results with reference to study objectives	9
Limitations	19	Discuss limitations of the study, taking into account sources of potential bias or imprecision. Discuss both direction and magnitude of any potential bias	9
Interpretation	20	Give a cautious overall interpretation of results considering objectives, limitations, multiplicity of analyses, results from similar studies, and other relevant evidence	10-12
Generalisability	21	Discuss the generalisability (external validity) of the study results	11-12
**Other information**			
Funding	22	Give the source of funding and the role of the funders for the present study and, if applicable, for the original study on which the present article is based	13

Table S1. Results from mixed-effects regression analyses on monthly trends

Dependent variable	AUDIT-C sum score†		% of patients scoring 8+ on AUDIT-C‡	
Country	Colombia	Mexico	Colombia	Mexico
N	10,658	6,613	10,444	6,569
Intercept	1.77<br>(1.46 to 2.15)***	1.96<br>(1.68 to 2.30)***	0.02<br>(0.01 to 0.03)***	0.01<br>(0.01 to 0.03)***
Monthly trend§	0.985<br>(0.979 to 0.992)***	0.981<br>(0.975 to 0.986)***	0.89<br>(0.85 to 0.94)***	0.98<br>(0.96 to 1.01)
Provider sex (men = 1; women = 0)	1.29<br>(0.99 to 1.68)	1.09<br>(0.93 to 1.28)	0.79<br>(0.29 to 2.14)	1.50<br>(0.82 to 2.74)
Provider age (reference = 17 to 29)				
30-39	1.08<br>(0.84 to 1.39)	1.11<br>(0.93 to 1.32)	0.56<br>(0.20 to 1.64)	1.08<br>(0.53 to 2.16)
40-49	0.77<br>(0.58 to 1.04)	1.10<br>(0.90 to 1.35)	0.42<br>(0.13 to 1.32)	1.98<br>(0.93 to 4.21)
50-59	1.08<br>(0.67 to 1.74)	1.00<br>(0.76 to 1.32)	0.22<br>(0.02 to 3.02)	1.20<br>(0.41 to 3.57)
60-69	0.52<br>(0.30 to 0.89)*	1.04<br>(0.77 to 1.41)	0.68<br>(0.10 to 4.60)	0.81<br>(0.24 to 2.73)
70+	NA	0.93<br>(0.42 to 2.04)	NA	2.70<br>(0.19 to 39.12)
Not reported	1.50<br>(0.74 to 3.05)	1.02<br>(0.72 to 1.43)	NA	NA
Provider profession (reference = Doctor)				
Nurse (technician)	1.57<br>(1.25 to 1.98)***	1.04<br>(0.69 to 1.59)	2.28<br>(0.93 to 5.63)	3.35<br>(0.74 to 15.16)
Midwife or social worker	1.56<br>(0.67 to 3.60)	1.45<br>(0.86 to 2.42)	7.39<br>(0.45 to 121.16)	NA
Psychologist	1.29<br>(0.71 to 2.35)	0.98<br>(0.81 to 1.17)	9.71<br>(0.63 to 150.51)	1.23<br>(0.61 to 2.52)
Other / not reported	0.85<br>(0.52 to 1.41)	0.85<br>(0.70 to 1.05)	NA	0.56<br>(0.23 to 1.39)
Patient sex (reference = women)				
Men	2.04<br>(1.95 to 2.14)***	2.10<br>(2.00 to 2.21)***	7.83<br>(5.94 to 10.31)***	4.99<br>(3.60 to 6.92)***
Not reported	1.19<br>(1.02 to 1.38)*	1.36<br>(1.00 to 1.84)*	1.18<br>(0.36 to 3.90)	NA‖
Patient age (reference = 18 to 29)				
30-39	0.98<br>(0.92 to 1.04)	0.92<br>(0.86 to 0.98)*	0.77<br>(0.55 to 1.09)	0.96<br>(0.63 to 1.44)

40-49	0.90 (0.85 to 0.96)**	0.84 (0.78 to 0.90)***	0.75 (0.52 to 1.01)	0.98 (0.65 to 1.47)
50-59	0.75 (0.70 to 0.80)***	0.66 (0.61 to 0.72)***	0.66 (0.44 to 1.01)	0.56 (0.34 to 0.90)*
60-69	0.56 (0.52 to 0.61)***	0.53 (0.48 to 0.58)***	0.24 (0.13 to 0.42)***	0.36 (0.19 to 0.68)**
70+	0.27 (0.24 to 0.30)***	0.38 (0.33 to 0.44)***	0.11 (0.05 to 0.26)***	0.13 (0.04 to 0.44)***
Not reported	0.92 (0.79 to 1.07)	0.69 (0.51 to 0.93)*	NA	NA
Patient education (reference = Less than high school)				
High school	1.25 (1.19 to 1.31)***	1.04 (0.98 to 1.10)	0.92 (0.68 to 1.24)	0.72 (0.45 to 1.04)
Beyond high school	1.16 (1.06 to 1.26)***	1.13 (1.06 to 1.21)***	0.73 (0.45 to 1.20)	0.80 (0.53 to 1.19)
Not reported	1.24 (1.08 to 1.42)**	1.01 (0.78 to 1.3)	0.73 (0.30 to 1.75)	0.86 (0.19 to 3.90)

Note. * $P \leq .05$; ** $P \leq .01$; *** $P \leq .001$

[†]Negative binomial mixed-effects regression analyses, with random intercepts for providers clustered within primary health care practices. Presented are Incidence Rate Ratios, i.e. exponentiated coefficients. Numbers in brackets indicate Wald-based confidence intervals.

[‡]Logistic mixed-effects regression analyses, with random intercepts for providers clustered within primary health care practices. Presented are Odds Ratios, i.e. exponentiated coefficients. Numbers in brackets indicate Wald-based confidence intervals.

[§]centered at beginning of COVID-19 pandemic.

[‖]N=44 cases were excluded from the regression model because none of them scored 8+ on the AUDIT-C resulting in singularity issues.

Abbreviation: AUDIT-C = Alcohol Use Disorder Identification Test – Consumption

Table S2. Results from mixed-effects regression analyses on period effect

Dependent variable	AUDIT-C sum score[†]		% of patients scoring 8+ on AUDIT-C[‡]	
Country	Colombia	Mexico	Colombia	Mexico
N	10,658	6,613	10,444	6,569
Intercept	1.92 (1.58 to 2.33)***	2.09 (1.79 to 2.45)***	0.02 (0.01 to 0.05)***	0.02 (0.01 to 0.03)***
Period effect (Reference period: data collected prior the pandemic onset)	0.79 (0.76 to 0.84)***	0.86 (0.80 to 0.92)***	0.41 (0.26 to 0.64)***	0.81 (0.55 to 1.20)
Provider sex (men = 1; women = 0)	1.31 (1.00 to 1.70)*	1.06 (0.91 to 1.23)	0.80 (0.29 to 2.21)	1.48 (0.81 to 2.71)
Provider age (reference = 17 to 29)				
30-39	1.08 (0.84 to 1.38)	1.09 (0.91 to 1.29)	0.52 (0.18 to 1.53)	1.07 (0.53 to 2.15)
40-49	0.77 (0.57 to 1.02)	1.10 (0.90 to 1.33)	0.36 (0.11 to 1.15)	1.99 (0.94 to 4.21)
50-59	1.07 (0.67 to 1.72)	0.98 (0.76 to 1.28)	0.24 (0.02 to 3.33)	1.20 (0.41 to 3.54)
60-69	0.53 (0.31 to 0.90)*	1.04 (0.77 to 1.39)	0.70 (0.10 to 4.85)	0.81 (0.24 to 2.72)
70+	NA	0.91 (0.42 to 1.98)	NA	2.66 (0.18 to 38.70)
Not reported	1.42 (0.70 to 2.87)	0.96 (0.69 to 1.33)	NA	NA
Provider profession (reference = Doctor)				
Nurse (technician)	1.59 (1.26 to 2.00)***	1.07 (0.71 to 1.63)	2.53 (1.02 to 6.26)*	3.39 (0.75 to 15.31)
Midwife or social worker	1.60 (0.69 to 3.67)	1.37 (0.83 to 2.25)	9.66 (0.58 to 162.34)	NA
Psychologist	1.25 (0.69 to 2.27)	0.93 (0.78 to 1.11)	9.71 (0.61 to 155.83)	1.23 (0.61 to 2.50)
Other / not reported	0.85 (0.52 to 1.39)	0.87 (0.71 to 1.06)	NA	0.56 (0.23 to 1.39)
Patient sex (reference = women)				
Men	2.04 (1.94 to 2.13)***	2.10 (2.00 to 2.21)***	7.85 (5.96 to 10.35)***	5.00 (3.61 to 6.94)***
Not reported	1.20 (1.03 to 1.50)*	1.33 (0.98 to 1.80)	1.24 (0.38 to 4.12)	NA[§]
Patient age (reference = 18 to 29)				

30-39	0.97 (0.92 to 1.03)	0.92 (0.86 to 0.99)*	0.76 (0.54 to 1.07)	0.96 (0.63 to 1.45)
40-49	0.90 (0.85 to 0.96)***	0.83 (0.78 to 0.90)***	0.75 (0.52 to 1.09)	0.98 (0.65 to 1.47)
50-59	0.74 (0.70 to 0.79)***	0.66 (0.61 to 0.71)***	0.65 (0.43 to 0.99)*	0.56 (0.34 to 0.90)*
60-69	0.56 (0.52 to 0.60)***	0.53 (0.48 to 0.58)***	0.23 (0.13 to 0.41)***	0.36 (0.19 to 0.68)**
70+	0.27 (0.24 to 0.29)***	0.38 (0.33 to 0.44)***	0.11 (0.05 to 0.25)***	0.13 (0.04 to 0.44)***
Not reported	0.90 (0.77 to 1.05)	0.69 (0.51 to 0.94)*	NA	NA
Patient education (reference = Less than high school)				
High school	1.25 (1.19 to 1.31)***	1.04 (0.98 to 1.10)	0.91 (0.68 to 1.24)	0.72 (0.45 to 1.04)
Beyond high school	1.16 (1.06 to 1.26)***	1.12 (1.05 to 1.20)***	0.73 (0.45 to 1.19)	0.78 (0.53 to 1.19)
Not reported	1.23 (1.08 to 1.41)**	1.00 (0.78 to 1.29)	0.71 (0.30 to 1.71)	0.86 (0.19 to 3.87)

Note. * $P \leq .05$; ** $P \leq .01$; *** $P \leq .001$

†Negative binomial mixed-effects regression analyses, with random intercepts for providers clustered within primary health care practices. Presented are Incidence Rate Ratios, i.e. exponentiated coefficients. Numbers in brackets indicate Wald-based confidence intervals.

‡Logistic mixed-effects regression analyses, with random intercepts for providers clustered within primary health care practices. Presented are Odds Ratios, i.e. exponentiated coefficients. Numbers in brackets indicate Wald-based confidence intervals.

§N=44 cases were excluded from the regression model because none of them scored 8+ on the AUDIT-C resulting in singularity issues.

Abbreviation: AUDIT-C = Alcohol Use Disorder Identification Test – Consumption

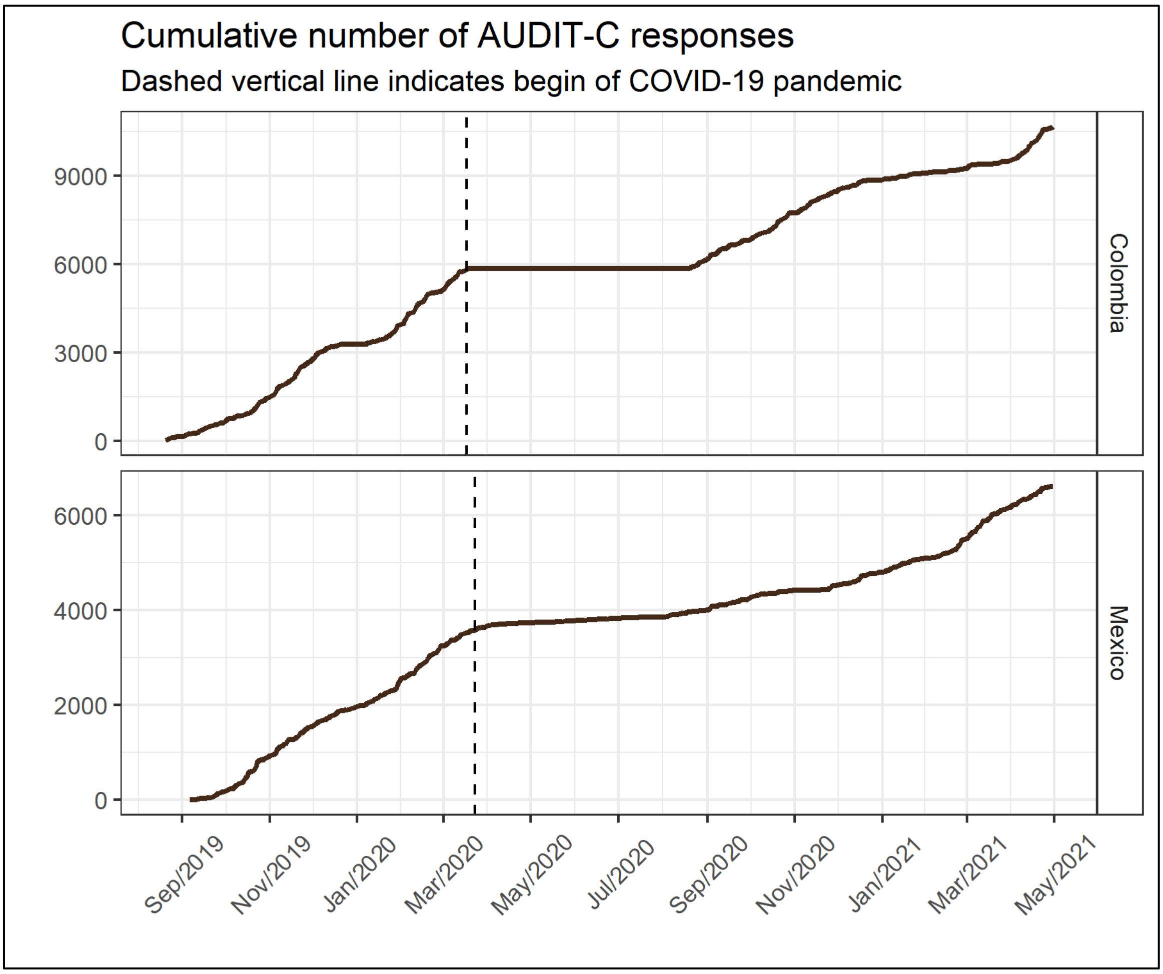

Appendix Figure 1. Cumulative number of AUDIT-C responses collected from primary health care patients in Colombia and Mexico. Dashed vertical lines indicate onset in COVID-19 pandemic in Colombia (17 March, 2020) and Mexico (March 23, 2020).

Abbreviation: AUDIT-C = Alcohol Use Disorder Identification Test – Consumption

Appendix Figure 2 illustrates the distribution of AUDIT-C scores among drinking patients (AUDIT-C score > 0) across the study period. In Colombia, available data from the pandemic show an overall decline in the median AUDIT-C score, with more than half of all consulting patients reporting to abstain from alcohol altogether (AUDIT-C score = 0). The share of patients scoring 8+ on the AUDIT-C or more ranged between 8 and 15% in the months August to December 2019 and declined to about 3% in January to March 2020. In ten out of eleven months during the COVID-19 pandemic (August 2020 to April 2021), less than 3% patients reported heavy drinking levels.

In Mexico, the share of heavy drinking patients was less variable in the pre-pandemic period (September 2019 and February 2020) and ranged between 4.0 and 6.1%. In the following months, the variation increased and both considerably lower (0% in July 2020) and higher (9.4% in June 2020) heavy drinking prevalence rates were recorded.

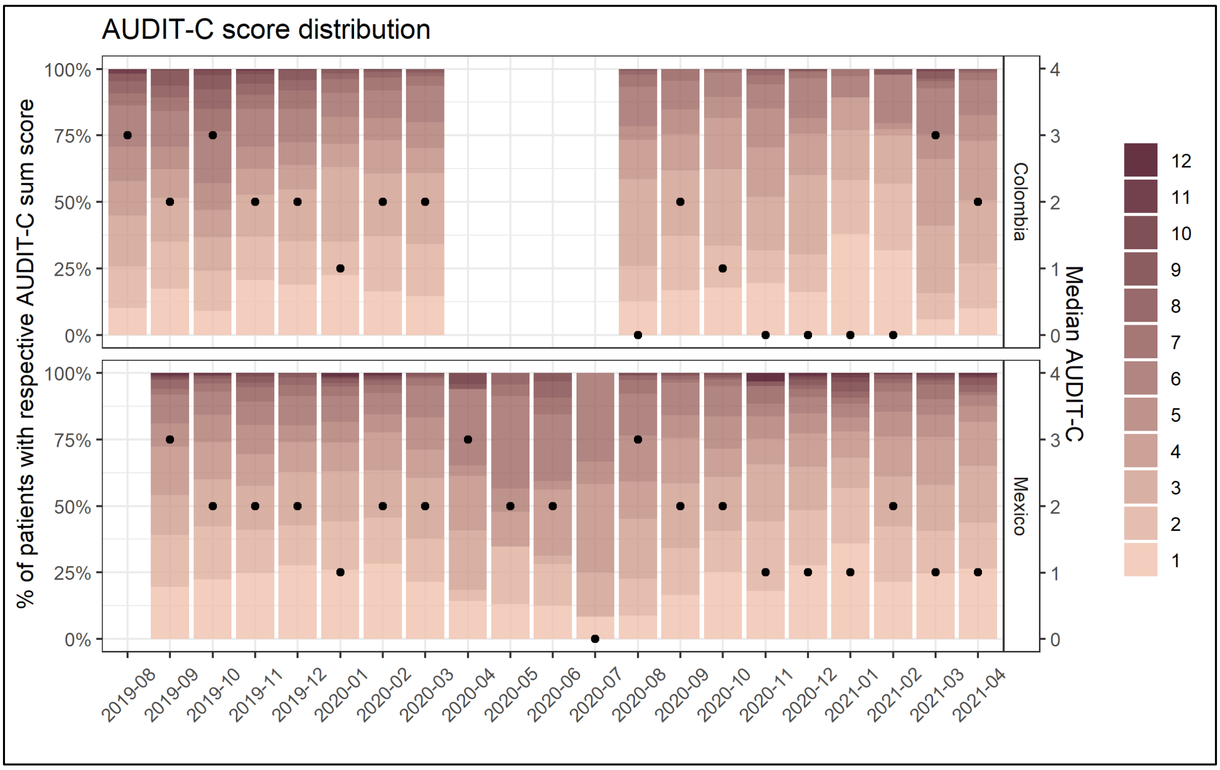

Appendix Figure 2. Distribution of the AUDIT-C score among primary health care patients documented by consulting providers in Mexico and Colombia

Abbreviation: AUDIT-C = Alcohol Use Disorder Identification Test – Consumption
